# Spectral separability and mapping potential of cassava leaf damage symptoms caused by whiteflies (Bemisia tabaci)

**DOI:** 10.1002/ps.4718

**Published:** 2017-10-24

**Authors:** Neil C Sims, Paul De Barro, Glenn J Newnham, Andrew Kalyebi, Sarina Macfadyen, Tim J Malthus

**Affiliations:** ^1^ CSIRO Land & Water Clayton Victoria Australia; ^2^ CSIRO Health & Biosecurity Dutton Park Queensland Australia; ^3^ National Crops Resources Research Institute Kampala Uganda; ^4^ CSIRO Agriculture & Food Black Mountain ACT Australia; ^5^ CSIRO Oceans & Atmosphere Dutton Park Queensland Australia

**Keywords:** spectroradiometer, remote sensing, disease mapping, Manihot, biosecurity, eastern Africa

## Abstract

**BACKGROUND:**

This study examines whether leaf spectra can be used to measure damage to cassava plants from whitefly (Bemisia tabaci), and the potential to translate measurements from leaf to landscape scale in eastern Africa. Symptoms of the cassava brown streak disease (CBSD) and cassava mosaic disease (CMD) viruses, and sooty mould (SM) blackening of lower leaves from whiteflies feeding on the upper leaves, were measured at the leaf scale with a high‐resolution spectroradiometer and a single photon avalanche diode (SPAD) meter, which retrieves relative chlorophyll concentration. Spectral measurements were compared to the five‐level visual scores used to assess the severity of each of the three damaging agents in the field, and also to leaf chemistry data.

**RESULTS:**

Leaves exhibiting severe CBSD and CMD were spectrally indistinguishable from leaves without any symptoms. Severe SM was spectrally distinctive but is likely to be difficult to map because of its occurrence in the lower crown. SPAD measurements were highly correlated with most foliar chemistry measurements and field scores of disease severity. Regression models between simulated Sentinel 2 bands, field scores and SPAD measurements were strongest using wavelengths with high importance weightings in random forest models.

**CONCLUSION:**

SPAD measurements are highly correlated to many foliar chemistry parameters, and should be considered for use in mapping disease severity over larger areas. Remaining challenges for mapping relate to the subtle expression of symptoms, the spatial distribution of disease severity within fields, and the small size and complex structure of the cassava fields themselves. © 2017 The Authors. *Pest Management Science* published by John Wiley & Sons Ltd on behalf of Society of Chemical Industry.

## 1 INTRODUCTION

Increasing global populations and potential impacts of climate change have reinvigorated interest in global food security, which is defined by the United Nations Food and Agriculture Organisation as existing when “…all people, at all times, have physical, social and economic access to sufficient, safe and nutritious food which meets their dietary needs and food preferences for an active and healthy life”. Increases in yields through plant breeding, increases in cropping intensity and expansion of arable land are fundamental to ensuring sufficient food supply in developing economies such as those in many African nations. However, sustaining yields through vigilance in biosecurity remains a key challenge globally.

Of particular interest in the East African region has been the impact of the invertebrate pest cassava whitefly (*Bemisia tabaci*) on cassava yield and food quality. Cassava is one of the main sources of nutrition in humid and subhumid tropical regions globally, including Malawi, Uganda and Tanzania. In these countries, cassava is an especially important crop for smallholder farmers and their families as it is drought tolerant and can be grown successfully in a range of soil types^1^, ^2^. Cassava leaves are used for a variety of purposes, but it is the tuberous root harvested after about 12 months of growth that is an important source of calories.

*Bemisia tabaci* is a sucking pest species complex 3 that is responsible for transmitting the viruses that cause a large number of plant damage symptoms throughout the East African region. These include cassava brown streak virus disease (CBSD),^4^ which causes brown lesions on the stem and in the tubers, making the crop unpalatable to animals or livestock, and cassava mosaic disease (CMD),^5^ which causes foliar chlorosis and leaf curling. Both CBSD and CMD can significantly reduce crop yields. These viruses can also be transmitted through the use of infected cuttings for the next season's planting. A third damaging agent; sooty mould (SM), has arisen because of a substantially increased abundance of *B. tabaci* in cassava production landscapes since the 1990s. The cause of increased *B. tabaci* abundance remains uncertain^6^, but increased numbers of *B. tabaci* have also led to an increase in direct feeding damage leading to leaf necrosis, and a higher prevalence of SM as a result of the deposition of honeydew from feeding *B. tabaci* onto lower leaves, which reduces photosynthesis 7.

Remote sensing surveillance methods uniquely provide an opportunity to monitor large regions in fine spatial detail, which is critical for effective biosecurity surveillance, and at a temporal frequency that is otherwise difficult to achieve. Large‐scale remote sensing initiatives that can benefit developing economies include Spurring a Transformation for Agriculture through Remote Sensing (STARS), which aims to implement remote sensing technologies for smallholder agricultural systems in emerging economies (http://www.stars‐project.org/en/contact/faq/), and the Group on Earth Observations Global Agricultural Monitoring (GeoGLAM) project, which aims to improve the use of remote sensing in crop projections and weather forecasting globally (https://www.earthobservations.org/geoglam.php). To date, however, no studies have used remote sensing data to examine the damage caused by *B. tabaci* to cassava crops in eastern Africa.

A key element in these initiatives is linking remote sensing observations with the appropriate field data. This is a critical step in calibrating models of plant pest and disease distribution and severity, and validating predictions. The current best practice in assessing CBSD, CMD and SM is to assess the severity of the diseases visually using a five‐level scale, with 1 being unaffected and 5 being severely affected. It is not clear, however, whether this field scoring method is suitable for comparison against remotely sensed observations for the purpose of classifying disease severity across larger areas.

This study examined the spectral effects of symptoms of CBSD, CMD and SM on cassava crops. We ask, first, whether the disease symptoms can be distinguished from one another, and from healthy plants, using leaf‐scale spectral reflectance data. Second, we examined whether the current field assessment method represents disease severity in a way that is suitable for use in remote sensing investigations. Third, we discuss the implications of this study for biosecurity surveillance with satellite remote sensing in other landscapes, most notably the potential for addressing issues in eastern Africa.

### 1.1 Pathology

**Figure 1:**
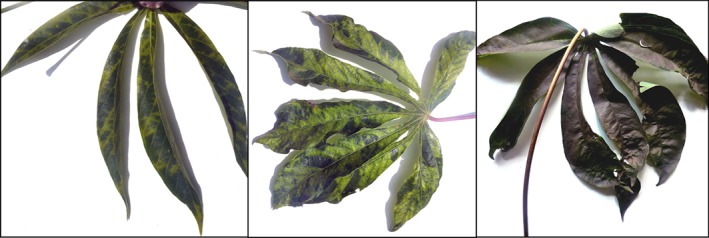
Leaf symptoms of severe infection for CBSD (left), CMD (centre) and SM (right).

Of the two viruses being investigated in this study, the most serious is CBSD which makes the cassava crop unsuitable for consumption by humans and livestock. The most diagnostic symptom of CBSD is the appearance of rough brown streaks along the stems at moderate infection levels^8^. Leaf symptoms at lower severity levels are typically subtle, and can include small patches of moderate chlorosis adjacent to minor veins on mature leaves or localised around the sites of whitefly feeding (Figure 1, left), with complete defoliation occurring in severe cases.

CMD is characterised by widespread and severe chlorosis and leaf curling (Figure 1, centre). Levels of CMD infection from moderate to severe typically reduce plant photosynthetic performance which, if prolonged, reduces crop yield. CMD infection is not always terminal for cassava plants, but losses caused by CMD infection are thought to cost around $2 billion in eastern Africa per year^9^.

In addition to transmission by whiteflies, the CBSD and CMD viruses may also be spread by the planting of infected stem cuttings. Whereas the symptoms of infection from whitefly transmission may develop over time in plants that are initially healthy, plants grown from infected stem cuttings exhibit leaf symptoms from the first leaf emergence.

SM is caused by the sugary honey dew, excreted by feeding whiteflies, dropping onto leaves lower in the canopy (Figure 1, right). The honeydew provides habitat substrate for saprophytic fungi which give the appearance of the leaves being covered with soot^10^. SM reduces photosynthesis and leaf transpiration, which in turn reduces plant performance and yield.

Many of the cassava varieties sampled were bred to be resistant to a specific disease and therefore showed negligible symptoms for one or more of the damaging agents, but in certain cases breeding for immunity to one disease may increase vulnerability to another. Conversely, some plants may exhibit symptoms of all of the damaging agents. The distribution of visible symptoms of these diseases may also be highly variable, including between neighbouring plants of the same variety.

## 2 Methods

### 2.1 Field data collection

The study was conducted in September 2015 in the cassava field trial plantations on the grounds of the National Crop Resources Research Institute (NaCRRI) at Namulonge, some 25 km north of Kampala, Uganda. The field trials were used to assess the yield and disease resistance of new cassava varieties that are bred for wider distribution to cassava growers in the region. The field trial areas consisted of small fields (approximately ¼ ha in size), each containing stands of one or more cassava varieties, surrounded by forest or other crop types. This complex mosaic of small cassava stands in a variable background is typical of how cassava is grown throughout much of the East African region.

Field assessments of the severity of CBSD, CMD and SM on each plant were conducted visually by one expert surveyor from NaCRRI using their standard five‐level scale. The visual severity scores were cross‐checked by another experienced surveyor at NaCRRI and found to be sufficiently consistent between assessors, with no systematic bias evident. As a consequence of the co‐occurrence of symptoms, a plant may have a CBSD score of 4 (heavily infected), a CMD score of 1 (not infected) and an SM score of 3, for example.

Sampling locations and plants were selected to encounter the widest possible range of CBSD, CMD and SM symptoms on the NaCRRI site. Spectral reflectance values, symptom scores and cassava variety information were recorded for 20 cassava plants across 10 cassava varieties (Table 1), yielding a total of 113 leaf spectra. Leaf position was recorded as upper, mid or lower canopy, and all leaves measured in this study were mature. Photographs of every leaf sampled were taken against a painted panel background featuring a white centre and black edges to aid image calibration.

**Table 1:** Sample size per severity class per damaging agent (n = 113). Varieties sampled were nam130, nase1, nase3, nase4, nase12, nase13, nase14, tme204, tp24 and ug120149

	Severity class				
	1	2	3	4	5
CBSD	62	12	15	6	18
CMD	80	3	15	0	15
SM	83	0	0	0	30

Spectral measurements were collected using two instruments. The primary instrument was an Analytical Spectral Devices (ASD, Longmont, CO, USA) FieldSpec Pro Hi‐Res spectrophotometer which measures leaf reflectance at wavelengths between 350 and 2500 nm in 3‐nm increments from 350 to 1000 nm, and in 8‐nm increments from 1000 to 2500 nm, and resamples the spectra in approximately 1‐nm increments during export. Leaf reflectance was measured using a leaf clip in conjunction with a calibrated light source and an embedded white panel as the background. The leaf clip measures reflectance over an area of about 2.5 cm x 2.5 cm, and provides consistent illumination and measurement conditions to ensure comparability of spectra between individual leaf samples. Measurements were also taken with a Konica‐Minolta (Tokyo, Japan) Single Photon Avalanche Diode (SPAD) 502 chlorophyll meter, which uses transmission at 650 and 940 nm to retrieve relative chlorophyll concentration (in SPAD units). Three SPAD and three spectroradiometer measurements were collected from each leaf and the spectra were averaged before analysis.

Wet chemistry analysis was conducted at NaCRRI for 13 plants including healthy leaves and those exhibiting severe or distinctive symptoms. These analyses retrieved moisture content and chlorophyll a, chlorophyll b, carotenoid and anthocyanin concentrations. Chlorophyll a is the most important and common biomolecule for photosynthesis, and chlorophyll b is the main accessory pigment. High chlorophyll concentrations typically indicate healthy plant functioning. The ratio of chlorophyll a to chlorophyll b, and the total chlorophyll concentration were also calculated from these data.

Carotenoids, along with chlorophyll, are the main leaf pigments in healthy green leaves − they absorb light for photosynthesis and protect the chlorophyll from sun damage. Carotenoids are typically yellow or red in colour and changes in leaf carotenoid concentration, and the proportion of carotenoids relative to chlorophyll, are widely used for diagnosing the physiological state of plants^11^. Anthocyanins also protect leaves from sun damage and may be red, purple or blue in colour^12^. High carotenoid or anthocyanin concentrations may indicate a diminished photosynthetic capability or a response to plant stresses including herbivory. Low leaf moisture concentration leads to the loss of leaf internal structure and can be interpreted as a symptom of poor plant performance, as lower evapotranspiration levels are also associated with lower crop yield^13^.

### 2.2 Spectral data analysis

Preliminary analysis showed that the spectral signatures of healthy leaves were sufficiently similar between varieties to avoid the need to stratify the dataset based on variety. In addition, the unique resistance characteristics of some of the varieties meant that the most severe expressions of certain diseases were exhibited in only one variety. For example, severe SM score 5 was observed only in the nam130 variety, but the spectra of nam130 plants not affected by any damaging agents (field score of 1 for all diseases) were indistinguishable from those of other varieties with severity scores of 1 for all diseases. Spectra were therefore more representative of symptom severity levels than differences between cassava varieties.

While no image data were acquired for this project, the potential to spectrally separate and identify these diseases in imagery was examined in two main ways. First, a range of spectral indices, which compare reflectance between two or more spectral regions sensitive to plant disease or stress symptoms, were calculated from the spectroradiometer measurements (Table 2). These indices were selected to identify different aspects of plant physiological functioning or photosynthetic processes, and also to match the range of data calculated from the samples in the leaf chemistry analysis. The modified chlorophyll absorption ratio index (MCARI)^14^ is sensitive to the relative abundance of chlorophyll. The photochemical reflectance index (PRI)^15^ targets light use efficiency, and the carotenoid reflectance index (CRI1)^11^ indicates the concentration of carotenoids relative to chlorophyll, which can be interpreted as a measure of plant stress. These indices use specific narrow wavelength bands that are best calculated from the narrow range and discrete wavelength reflectance data provided by the spectroradiometer.

**Table 2:** Spectral indices calculated from the reflectance (ρ) data

Index	Formula	Name	Reference
NDVI^a^	$\frac{\mathrm{NIR}-\mathrm{red}}{\mathrm{NIR}+\mathrm{red}}$	Normalised difference vegetation index	Tucker 16
EVI2^a^	$2.5\frac{\mathrm{NIR}-\mathrm{red}}{\mathrm{NIR}+2.4\;*\;\mathrm{red}+1}$	Two‐band enhanced vegetation index	Jiang et al. 17
MCARI	$\left[\left(\rho 700-\rho 670\right)-0.2\left(\rho 700-\rho 550\right)\right]*\left(\frac{\rho 700}{\rho 670}\right)$	Modified chlorophyll absorption ratio index	Daughtry et al. 14
PRI	$\frac{\rho 531-\rho 570}{\rho 531+\rho 570}$	Photochemical reflectance index	Gamon et al. 15
ARI	$\left(\frac{1}{\rho 550}\right)-\left(\frac{1}{\rho 700}\right)$	Anthocyanin reflectance index	Gitelson et al. 18
CRI1	$\left(\frac{1}{\rho 510}\right)-\left(\frac{1}{\rho 550}\right)$	Carotenoid reflectance index 1	Gitelson et al. 11

Wavelengths are in nanometres.

a Simulated Sentinel 2 bands: red = mean(ρ650 − 680); NIR = mean(ρ773 − 793).

NIR, near‐infrared.

A second group of indices included the normalised difference vegetation index (NDVI)^16^ and two‐band enhanced vegetation index (EVI2)^17^ which have been extensively used to assess plant productivity performance. These indices are often calculated from the broad‐band data that are available from a wide range of satellite image sources, in which the image bands show the average reflectance over a range of wavelengths around a band centre.

When simulating broad‐band data from the narrow‐band spectroradiometer data, we averaged reflectance over wavelength ranges to match the Sentinel 2 satellite MultiSpectral Instrument (MSI) bands (https://earth.esa.int/web/sentinel/technical‐guides/sentinel‐2‐msi/msi‐instrument). The Sentinel 2 MSI collects data in 13 spectral bands between visible blue (443 nm band centre wavelength) to short‐wave infrared (2190 nm) at pixel sizes ranging from 10 to 20 m in the visible and near‐infrared (NIR) wavelengths, to 60 m in spectral regions of strong absorption. Other satellite data sources such as Worldview 3 provide higher spatial resolution with pixels as small as 0.31 m (http://www.satimagingcorp.com/satellite‐sensors/worldview‐3/), which may improve disease severity detection in small and complex fields at the plant scale. However, these images are typically high cost and do not include an archive of repeat coverage, which is essential for monitoring changes in plant conditions.

Sentinel 2 has advantages over most of the high‐resolution satellites including high image capture frequency (5‐day repeat rate with sister satellites 2A and 2B in orbit), an archive of historical images and free access to the image data. High‐frequency imagery can be combined with high spatial resolution data to rapidly detect changes in plant condition and reveal distribution patterns in finer detail^19^. Sentinel 2 is likely to be well suited to mapping disease emergence and distribution at large regional scales, and more readily available than higher resolution images for studies of this kind in the East African region in the near future.

By comparing wavelength bands from a single acquisition, spectral indices may reduce the influence of variations in illumination, the colour of background material and leaf structure on image pixel values, which can improve the accuracy of detecting leaf stress 20. This is less important for use with the spectroradiometer data collected under the very controlled conditions of the leaf clip, but indices can significantly improve the consistency of satellite images which show a large area, or are captured at different locations or times. Indices can reduce the effects of atmospheric transparency, topographic variations and sun brightness which can vary across the image or between images.

Correlation analyses were used to examine the relationship between the visual field scores of disease severity, leaf chemistry data, SPAD measurements and indices calculated from the spectral measurements. Spearman's rank correlation was used in comparisons with the ranked field disease severity scores, and Pearson's correlation was used otherwise.

Further exploration of the potential of spectral methods for detecting these diseases involved identifying the spectral regions most indicative of these symptoms using the randomForest package in R 21. Random forest analysis has been shown to be superior to linear, quadratic and penalised discriminant analysis when developing models with hyperspectral data because of its robustness to overfitting^22^, ^23^. Disease severity was classified into two classes: ‘affected’, which includes samples with disease severity scores of 2 or above, and ‘not affected’, which includes only samples with a field score of 1 for the disease of interest. The significance of spectral bands was assessed using the measures of variable importance calculated during the random forest modelling, as per Newnham et al. 24 Variable importance represents how much the prediction error increases when data for that variable are permuted while all others remain unchanged 21, 25.

Binary logistic regression (using the glm package in R) was used to assess the potential accuracy of simulated Sentinel 2 data for mapping CBSD, CMD and SM, and a linear model was used to test MCARI against disease severity and SPAD measurements. The models were calibrated on 70 randomly selected observations and validated on the remaining 43. A linear model between the predicted regression results and the independent variable of interest was then calculated, and the mean adjusted r ^2^ value over 100 iterations of this process is reported here.

## 3 RESULTS

### 3.1 Spectral symptoms of the damaging agents

**Figure 2:**
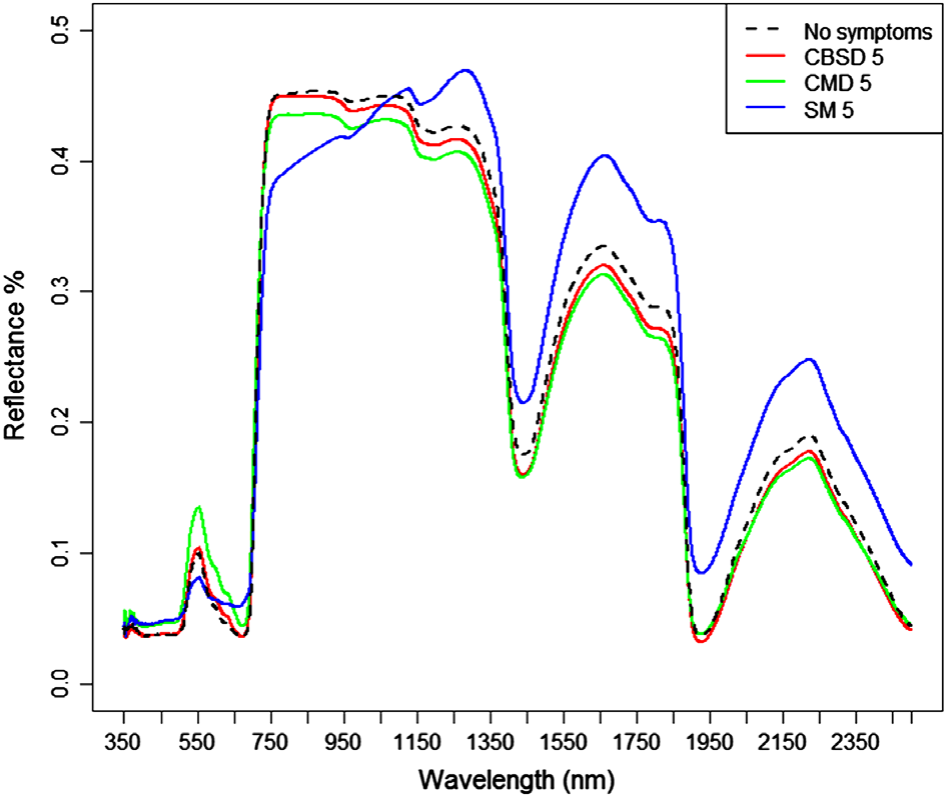
Mean spectra for leaves showing no disease symptoms (black, n = 8) versus severe CBSD (red, n = 18), CMD (green, n = 15) and SM (blue, n = 30).

Figure 2 shows the mean reflectance spectrum of unaffected leaves exhibiting no disease symptoms (field score 1 for all diseases – dashed black line) with that of leaves exhibiting severe symptoms (field score 5) for CBSD (red), CMD (green) and SM (blue) across all cassava varieties. The CBSD score 5 spectrum is virtually identical to that of the leaf showing no symptoms over the full spectral range, which is consistent with the generally subtle leaf symptoms of this disease. CMD score 5 spectra are also similar to those of the unaffected leaves over most of the spectral range, but CMD score 5 leaves exhibited higher reflectance in the visible green (550 nm) to red (650 nm) wavelengths than unaffected leaves, which is associated with the leaf yellowing that is characteristic of CMD infection.

The SM score 5 average spectrum has the most distinctive spectral response of these symptoms. Compared with the spectra of the other symptoms and leaves showing no symptoms, reflectance is more even across the visible wavelengths, with higher reflectance in the blue (450 nm) and red wavelengths, and reduced green reflectance. Reflectance is also substantially reduced in the near‐infrared between about 750 and 1150 nm, and generally higher at longer infrared wavelengths.

### 3.2 Correlation between spectral indices, field scores and laboratory chemistry measurements

**Figure 3:**
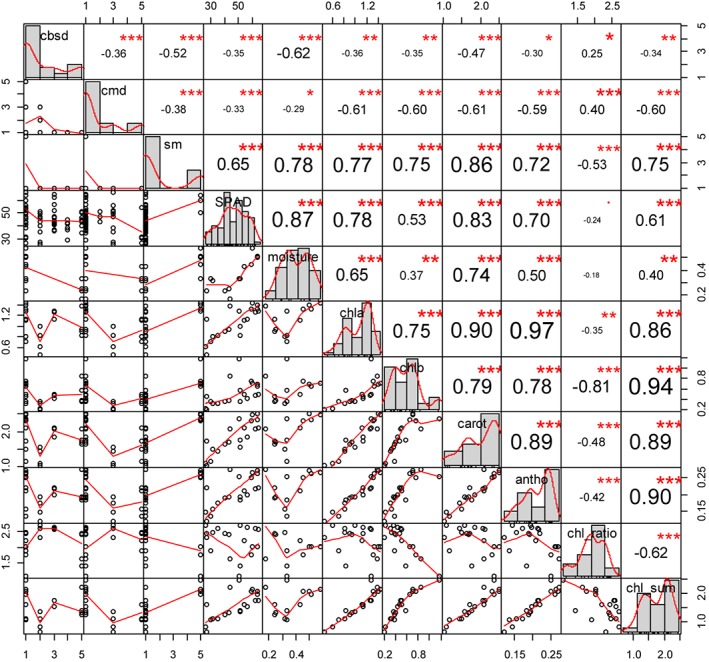
Spearman correlation (ρ) between field scores of disease severity and leaf chemistry. ***P < 0.0001; **P < 0.001; *P < 0.01. Variable names and their data distribution are shown along the diagonal. Numbers in the upper right indicate the correlation coefficient, with the size of the numerals increasing at higher correlation values. Plots in the lower left show the relationship between variable pairs at the intersection of the relevant row and column. Chla, chlorophyll a; chlb, chlorophyll b; carot, carotenoids; antho, anthocyanin; chl_ratio, ratio of chlorophyll a to chlorophyll b; chl sum, chlorophyll a plus chlorophyll b.

Figure 3 shows the Spearman rank correlation (*ρ*) between leaf chemistry data, SPAD measurements and visual disease severity scores. The *ρ* coefficient and significance are indicated in the upper right corner of the plots at the junction of the column and row of interest, and the data fit is shown in the lower left at the junction of the column and row of interest. The size of the numerals increases where the correlation is higher. This format is repeated for all correlation tables in this paper.

In general, leaf chemistry variables were highly inter‐correlated, which indicates that these damaging agents may induce a systemic reduction of photosynthetic functioning in cassava leaves. However, the presence of lower correlations suggests that each leaf chemistry variable also provides some unique information about these symptoms.

Amongst the visual field scores of diseases, SM was significantly correlated with more of the leaf chemistry results than the other diseases, most strongly with the concentration of carotenoids (*ρ* = 0.86). CBSD severity was most strongly correlated with moisture concentration (*ρ* = ‐0.62) and CMD was most strongly correlated with the concentrations of chlorophyll *a* and carotenoids (*ρ* = ‐0.61). Correlations of CBSD and CMD with the leaf chemistry were negative, indicating that increased severity was associated with reductions in leaf chemical concentrations, whereas correlations with SM were positive.

**Figure 4:**
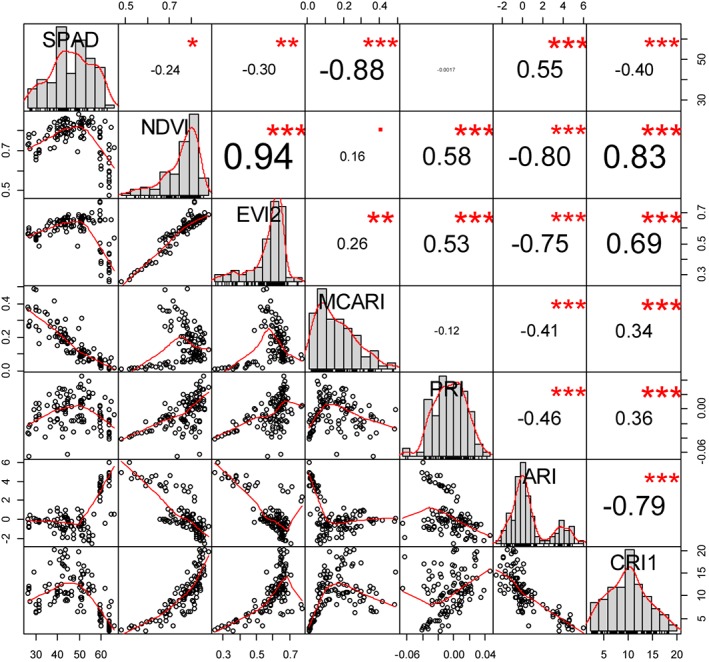
Pearson's correlation coefficient between SPAD measurements and spectral indices calculated from the reflectance data. ***P < 0.0001; **P < 0.001; *P < 0.01. Variable names and their data distribution are shown along the diagonal. Numbers in the upper right indicate the correlation coefficient, with the size of the numerals increasing at higher correlation values. Plots in the lower left show the relationship between variable pairs at the intersection of the relevant row and column.

The SPAD measurements were strongly and significantly correlated with the leaf chemistry data apart from the chlorophyll ratio. This suggests that SPAD measurements may be a useful alternative to the more expensive and time‐consuming leaf chemistry analyses. While the correlations between SPAD measurements and disease severity scores were highly significant (*P* < 0.0001), they were relatively weak except for SM, which is a binary metric in which only disease severity levels of 1 (not affected) or 5 (severely affected) were observed. While SM severity levels were statistically separable using the SPAD data (McFadden *r* ^2^ = 0.56; *P* < 0.001 for binomial logistic regression), the binary nature of the SM severity scores highlights a shortcoming of the visual field scoring method for accurately assessing intervals of disease severity in these plants. The SPAD measurements were highly correlated with all of the spectral indices (Figure 4) and most strongly with the MCARI (Pearson's *r* = ‐0.88).

### 3.3 Mapping potential

**Figure 5:**
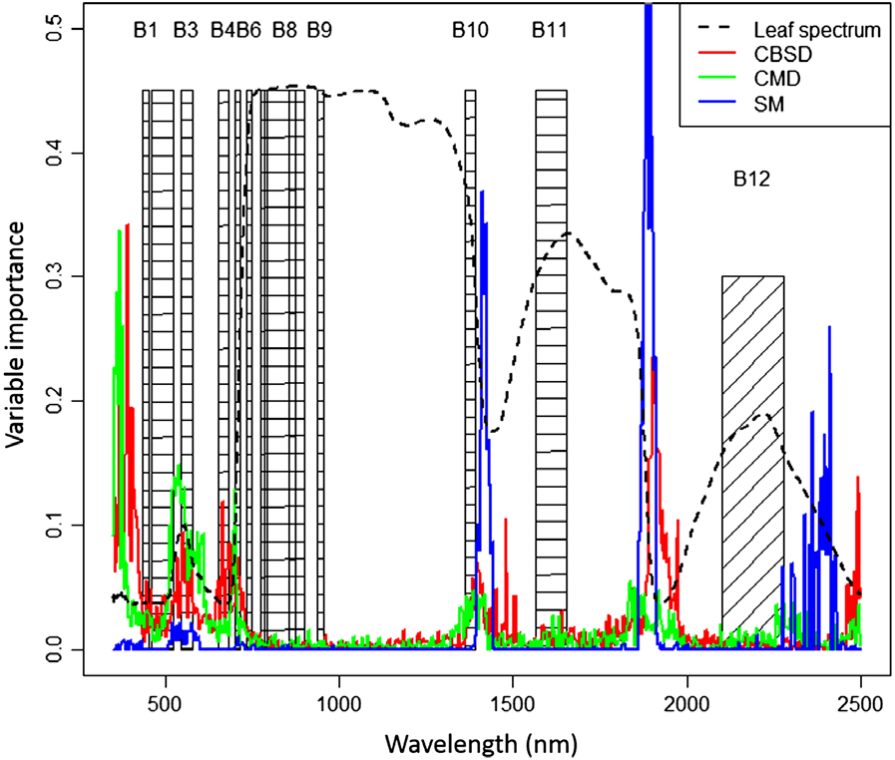
Sentinel 2 spectral bands (hashed regions) in relation to the variable importance measures and healthy leaf spectrum. Band numbers are shown at the top.

Figure 5 shows the variable importance scores for each disease calculated from a 500 model ensemble of random forest models between spectral data and each of the field scores, along with a spectrum from a cassava leaf that was not affected by any of the damaging agents (score 1 for all symptoms − dashed black line). Spectral regions of highest importance occurred in the visible and NIR wavelengths, and in regions of strong water absorption near 1400 and 1800 nm. The high importance of water absorption wavelengths for SM and CBSD reflects the stronger correlation between leaf moisture levels and the severity of these diseases than for CMD (Figure 3). Sentinel 2 bands (shown as hashed boxes in Figure 5) cover several of the regions of high variable importance in the visible and NIR wavelengths (bands 3, 4 and 5) and the water absorption feature around 1400 nm (band 10).

Binary logistic regression models were calculated between field scores of disease severity, SPAD measurements and three sets of simulated Sentinel 2 spectral bands including (1) bands 3, 4, 5 and 10, which occur in regions of high variable importance, (2) all Sentinel 2 bands and (3) the MCARI, which is calculated from Sentinel 2 MSI bands 3, 4 and 5 (Table 3). Predictive power was lowest for models of CBSD severity, with the strongest model calculated from bands in the regions of high variable importance (*r* ^*2*^ = 0.37; *P* < 0.0001). Models of CMD severity were strongest when using only the bands in the regions of high variable importance (*r* ^*2*^ = 0.48; *P* < 0.0001) and SM prediction was strongest when combining all simulated Sentinel 2 bands (*r* ^*2*^ = 0.77; *P* < 0.0001). SPAD measurements were best correlated with models of disease severity calculated from only the bands in the regions of high variable importance (*r* ^*2*^ = 0.84; *P* < 0.0001).

**Table 3:** Adjusted r ^2^ calculated from 100 iterations of a binary logistic regression model between simulated Sentinel 2 band combinations, field scores of disease severity and SPAD measurements

	CBSD	CMD	SM	SPAD
Sentinel 2, bands 3, 4, 5 and 10	0.37	0.48	0.65	0.84
Sentinel 2, all bands	0.24*	0.46*	0.77	0.80
Sentinel 2, MCARI	0.07^NS^	0.15*	0.37	0.75

All models are significant at *P* < 0.0001 except *P < 0.01 and ^NS^not significant

## 4 DISCUSSION

This is the first study to examine the potential of spectral reflectance measurements to identify and classify the severity of symptoms of three damaging agents caused by *B.tabaci* on cassava leaves in eastern Africa. This study addresses the spectral separability of the disease symptoms and severity levels, the suitability of the visual assessment scores of disease severity for comparison with remote sensing data and the potential to map the symptoms in satellite image datasets.

This study has included samples from 10 cassava varieties sampled in the NaCRRI field trial area that are currently being tested for distribution to growers in eastern Africa. We found no significant difference in the spectral characteristics of healthy foliage between those varieties, but these plants are a subset of the great many cassava varieties under cultivation in the East African region. Other varieties may exhibit variations in leaf colour which could alter the spectral separability from one another, or influence the visibility of foliar symptoms, and this study should not be considered representative of all cassava varieties.

Spectrally, amongst the varieties tested here, leaf symptoms of CBSD and CMD were similar to leaves showing no damage symptoms. This result, and their relatively weak correlation to either the foliar chemistry or the SPAD measurements, suggests that it is unlikely that these symptoms could be diagnosed from other damaging agents with similar leaf symptoms using spectral data alone. The symptoms of SM were more spectrally distinctive from those of the other diseases and from healthy foliage, but the likelihood of mapping SM severity is probably also low because SM occurs on the lower leaves which are less visible to overhead satellite or airborne sensors.

In terms of the impacts of these diseases on leaf physiology, correlations amongst the leaf chemistry data were generally high, indicating that these damaging agents may have a systemic impact on leaf functioning that affects many aspects of leaf condition. The correlation between leaf chemistry measurements and the severity of CBSD and CMD symptoms was negative (apart from the chlorophyll ratio), which indicates that increased severity of these diseases tends to reduce the concentration of leaf moisture and photochemicals. This is consistent with symptoms of a range of damaging agents that also cause foliar chlorosis or leaf curling such as cassava mealybug or cassava green mite, and also abiotic factors such as differences in moisture or nutrient availability.

The positive correlation between leaf chemistry variables and SM severity indicates a different physiological outcome from this damaging agent. Cassava leaves are generally intolerant of shade and, while shaded cassava leaves may have higher chlorophyll *a* concentrations than fully illuminated leaves^26^, they are typically adapted to a high light environment. Higher solar exposure tends to result in higher chlorophyll and moisture concentrations, and lower carotenoid and anthocyanin concentrations^27^. Leaves from which SM samples were collected were not cleaned of their SM coating as our aim was to simulate the condition of the leaves as they were likely to appear in imagery. Investigating the spectra of cleaned leaves in relation to SM severity may be useful for confirming the physiological changes in leaf functioning associated with this damaging agent in future.

These results indicate that the continuous representation of symptom severity from the SPAD meter is better correlated to the leaf chemistry, and may be better suited for comparison with satellite image data, than the categorical five‐level visual scores that are currently used. The relatively weak correlation between the SPAD measurements and field severity scores for CBSD (*r* = −0.35) and CMD (*r* = ‐0.33), however, suggests that these methods may be measuring different aspects of symptom expression. The NaCRRI staff who provided and checked the field scores are highly trained specialists who have relied on this visual assessment method to guide their breeding programmes and research practices for many years. These specialists integrate observations not only of foliage colour but also of the shape and form of the leaves, stems and the plants as a whole, which were not accounted for in this study. For correlation with actual disease severity, quantitative measurements of virus titre in leaf tissues may be required, but these options are currently not readily available in this region.

The field assessment of plant condition can be influenced by a range of factors. First, health assessments are somewhat subjective, even when conducted by experienced staff. While no systematic bias was found in our results, measurements by multiple staff could reduce the consistency of damage interpretations. At large scales, the interpretation of severity assessments at a given location may also be influenced by the severity of symptoms in the surrounding plants. A plant that may be scored a 3 (moderately affected) in a region of generally severe disease levels may be scored a 5 (severely affected) when surrounded by plants in generally good condition, for instance. The inclusion of SPAD measurements as part of the field assessment process could reduce errors from many of these sources and improve the correlation with broad‐scale satellite imagery.

This study also demonstrates the potential suitability of Sentinel 2 MSI data for mapping these symptoms across the East African region. Sentinel 2 MSI data include spectral bands located in regions of high variable importance as identified using random forest modelling, and they have a spatial resolution that is amongst the finest of the freely available satellite image data sources, with pixels as small as 10 m x 10 m in some bands. Potentially suitable higher spatial resolution imagery is available from a range of commercial space‐borne and airborne systems including Unmanned Aerial Vehicles (UAVs, or drones), which are being deployed for a range of smaller scale studies in this region by the STARS project. UAVs can be equipped with hyperspectral sensors providing image data in many wavelengths, similar to that of the ASD spectroradiometer used in this study. Because of the relatively low altitude of UAVs, UAV imagery is suited to assessment at the tree to field scale. UAV images can be challenging to use across large regions, however, because of the additional calibration complexity in creating mosaics of many images to obtain the required spatial coverage. These factors can increase processing costs and reduce map accuracy, and at large scales high‐resolution satellite imagery is likely to remain the most suitable and cost‐effective option.

Mapping these symptoms over large regions is further complicated, however, by spatial complexity at a range of scales. The cassava fields are typically small and in close proximity to other crops or native vegetation. Within fields, these damaging agents may appear as severely affected plants standing next to unaffected ones. There is also a lack of spatial information identifying the locations of cassava plantations in eastern Africa, which complicates the translation of disease models across large regions.

A monitoring approach in which changes in crop reflectance are assessed over time may be more effective than attempting to detect these diseases based on spectral data alone. This approach is likely to be most useful where the damaging agents are transmitted by the whiteflies and disease symptoms develop subsequently, rather than where the disease results from planting infected cuttings in which symptoms usually appear on the first leaves as they emerge. Sentinel 2's high capture frequency and image archive, which enable changes in condition to be monitored over short and longer time scales, may also be an advantage here.

## 5 CONCLUSION

This is the first study describing the leaf‐scale spectral symptoms of three damaging agents caused by *B.tabaci* on cassava leaves in eastern Africa. Our analysis has focused on the spectral characteristics of these damaging agents, the suitability of methods of assessing symptom severity in the field for use in remote sensing studies, and the potential to map these symptoms across the landscape. While a number of challenges remain in each of these areas, this study points to potential options for improving the detection, severity classification and mapping of whitefly damage symptoms. First, the accuracy of translating leaf‐scale disease assessments to the landscape scale can be improved by increasing the sensitivity of field assessments through the use of sensing equipment such as SPAD meters. Second, selecting image bands based on their variable importance calculated from random forest modelling can improve models of the symptom severity of these damaging agents. Third, the effectiveness of a monitoring approach using Sentinel 2 data to detect changes in plant condition over time should be assessed for mapping the distribution and severity of whitefly damage to cassava crops in this region. The strong dependence upon this crop for nutrition in the East African region should encourage further investigation of these issues in subsequent studies.
